# Association entre les hormones sexuelles, les marqueurs de remodelage osseux et la densité minérale osseuse chez des femmes ménopausées d'origine marocaine (étude transversale)

**DOI:** 10.11604/pamj.2015.22.106.5532

**Published:** 2015-10-07

**Authors:** Aissam El Maataoui, Asmae Biaz, Fatima El Boukhrissi, Si El Machtani, Abdellah Dami, Sanae Bouhsain, Youssef Bamou, Abdellah El Maghraoui, Zhor Ouzzif

**Affiliations:** 1 Université Mohamed V Souissi, Faculté de Médecine et de Pharmacie de Rabat, Laboratoire de Biochimie de l'Hôpital Militaire d’‘Instruction Mohamed V, Rabat, Maroc; 2 Laboratoire de Biochimie de l’‘Hôpital Militaire d'Instruction Mohamed V, Rabat, Maroc; 3 Laboratoire de Biochimie de l’‘Hôpital Militaire Moulay Ismail, Meknès, Maroc; 4 Université Mohamed V Souissi, Faculté de Médecine et de Pharmacie de Rabat, Service de Rhumatologie et de la Rééducation Fonctionnelle de l’‘Hhôpital Militaire d'Instruction Mohamed V, Maroc

**Keywords:** Hormones sexuelles, densité minérale osseuse, marqueurs de remodelage osseux, sex hormones, Bone mineral density, bone remodeling markers

## Abstract

**Introduction:**

Le présent travail se propose d’étudier la relation entre les hormones sexuelles, notamment l’œstradiol et l'indice de l’œstradiol libre, le sulfate de déhydroépiandrosterone et la sex hormone binding globulin, les marqueurs de remodelage osseux et la densité minérale osseuse chez une population de femmes marocaine ménopausées.

**Méthodes:**

Il s'agit d'une étude transversale, menée sur une période de 6 mois d'octobre 2012 à fin avril 2013 et ayant fait participer deux cent deux (202).

**Résultats:**

L’œstradiol et l'indice d’œstradiol libre (IEL) ont montré une corrélation négative respectivement à l'ostéocalcine (OC), à la crosslaps (β-CTX) et l'OC (p<0.001). La sulfate de déhydroepiandrosterone S-DHEA a été corrélée positivement à l'OC (p<0.001), alors que e taux sérique de la sex hormone binding globulin (SHBG) l'a été corrélé à la β-CTX et l'OC (p<0.001). Par ailleurs, une corrélation positive a été établie entre la densité minérale osseuse (DMO) au col de fémur et le poids, l'indice de masse corporelle (IMC), l'IEL et la S-DHEA. Une corrélation négative a été retrouvée entre la DMO au col de fémur d'une part et l’âge, la DDR, la SHBG, la β-CTX et l'OC d'une autre part.

**Conclusion:**

Le présent travail montre que l'augmentation de l’âge et la diminution de l’œstradiol libre expliquent la diminution de la DMO au niveau du col du fémur, alors que l'augmentation du taux sérique de la SHBG et la diminution du poids expliquent la diminution de la DMO au rachis lombaire.

## Introduction

Les stéroïdes sexuels jouent un rôle clé dans le développement et le maintien du capital osseux chez les hommes et les femmes. Les estrogènes ont, en effet, un rôle capital dans la régulation du métabolisme osseux chez la femme. Ainsi après la ménopause, la production endocrine ovarienne des estrogènes est remplacée par une production intracrine au niveau des tissus basés sur la conversion des androgènes d'origine surrénale [1]. Les estrogènes agissent directement et indirectement sur le tissu osseux. De façon indirecte, ils modulent la production et l'effet des hormones calciotropes (hormone parathyroïdienne, métabolites de la vitamine D) pour favoriser une concentration optimale de calcium au niveau des sites de minéralisation. Les estrogènes agissent également directement sur le tissu osseux où leur effet principal est d'inhiber l'activité ostéoclastique [1]. La déhydroépiandrostérone (DHEA) et son sulfate (S-DHEA) sont les stéroïdes circulants les plus abondants. Les taux plasmatiques de DHEAS (100-400 µg/dL) reflètent la production d'androgènes surrénaliens. Elles sont sécrétées par les glandes surrénales, diminuent avec l´âge, et ont été impliqués dans une variété de conditions physiopathologiques liées à l’âge y compris l´ostéoporose [2]. Plusieurs études ont examiné la relation entre la S-DHEA et l'ostéoporose, mais avec des résultats qui restent contradictoires. Ceci est peut être du à la taille des populations étudiées et au types d’études qui sont en général des études transversales [3–5]. La sex hormone-binding globulin (SHBG) est une glycoprotéine plasmatique qui lie avec une forte affinité les stéroïdes sexuels régulant ainsi leur biodisponibilité et en d'autres termes les taux des formes actives. Elle intervient dans de nombreuses situations pathologiques dont l'ostéoporose [6]. La SHBG possède son propre récepteur (R-SHBG) et exerce par son intermédiaire une action membranaire et intracellulaire, modulant ainsi le signal stéroïdien [6]. Les études sur les relations entre la concentration de la SHBG et la densité minérale osseuse sont moins nombreuses. Le but de la présente étude est de chercher une association entre les hormones sexuelles à savoir l’œstradiol et l'indice de l’œstradiol libre, la déhydroépiandrosterone sulfate ainsi que la sex hormone binding globulin, les marqueurs de remodelage osseux et la densité minérale osseuse chez une population de femmes marocaine ménopausées.

## Méthodes

### Patients

Il s'agit d'une étude transversale, menée sur une période de 6 mois, d'octobre 2012 à fin avril 2013. Quatre cent sept (407) femmes marocaines volontaires ménopausées ont formulé leur consentement éclairé pour participer à l’étude. Seules les femmes ayant répondu aux critères d'inclusion ont été intégrées. Il s'agit de femmes ménopausées de plus de 24 mois, sans antécédents de fractures d'origine ostéoporotiques, n'utilisant pas de suppléments hormonaux (traitement hormonal substitutif) ou de médicaments affectant la minéralisation osseuse, qui sont indemnes de pathologies interférant avec le métabolisme osseux comme les maladies thyroïdiennes et parathyroïdiennes, les hépatopathies chroniques et n'ayant pas eu de fracture dans les deux années précédentes.

### La mesure de la densité minérale osseuse (DMO)

La DMO a été mesurée chez toutes les patientes au niveau du rachis lombaire et du col fémoral par la technique d'Absorptiométrie biphotonique à rayons X (DXA) sur l'appareil Lunar prodigy vision, General Electric^®^, selon le même protocole et par la même équipe. La définition de l'OMS a été retenue pour classer les patientes en ostéoporotiques (T-score ≤ -2.5), ostéopéniques (-2.5 T-score<-1) et normales (T-score ≥ -1). La population de référence utilisée était constituée de jeunes femmes marocaines choisies au hasard dans la ville de Rabat. Au moment de l´étude, les mesures sur Phantom ont montré des résultats stables. La précision de Phantom exprimée par le coefficient de variation du pourcentage était de 0,08. De plus, la reproductibilité a été évaluée dans la pratique clinique et a montré une plus petite différence de 0,04 g/cm^2^ (Rachis lombaire) et 0,02 (hanches).

### Les paramètres biologiques

Les prélèvements sanguins requis pour la réalisation des différents examens ont été effectués à jeun par ponction veineuse, le matin entre 7h et 9h, dans des tubes sans anticoagulant (tubes secs). Les échantillons sanguins ont été rapidement acheminés au laboratoire et centrifugés pendent 15 minutes à 3000 tours/min. Les sérums décantés après centrifugation ont été aussitôt congelés à -70°C jusqu’ au moment de leur analyse. Le dosage de la β-crossLaps (β-CTX), de l'Ostéocalcine (OC), de la parathormone (PTHi), de la Sex hormone binding globulin (SHBG), de l’œstradiol (E2), de la déhydroepiandrosterone sulfate (S-DHEA) et de la 25 hydroxy-vitamine D3 (VITD) ont été réalisés sur l'auto-analyseur Cobas^®^ 6000 de la société Roche Diagnostics^®^, par Electrochimiluminescence en méthode « sandwich ». Nous tenons à préciser rapporter que les kits utilisés pour le dosage de la vitamine D dosent seulement la vitamine D3 et non un dosage de la vitamine D totale. L'indice de l’œstradiol libre (IEL) est calculé selon la formule suivante: E_2_(pmol/l) / SHBG(nmol/l).

### Analyses statistiques

Les résultats ont été exprimés par la moyenne ± l’écart type pour les variables continues et par le pourcentage (effectif) pour les variables discontinues. La distribution normale des variables continues a été vérifiée par le test Kolmogorov-Smirnov. L'analyse de la variance (ANOVA) a été utilisée pour la comparaison des variables continues entre groupes. Le coefficient de Pearson a été utilisé pour évaluer la corrélation des paramètres cliniques et biochimiques. La régression multiple a été effectuée pour identifier les paramètres prédictifs de la DMO au niveau du col de fémur et du rachis lombaire. Les données ont été traitées par les logiciels Excel 2007 et SPSS 15.0 pour Windows. Les résultats sont considérés statistiquement significatifs à partir d'une valeur de p≤ 0,05. L'indice de masse corporelle a été calculé avec la formule suivante: IMC = poids / (taille)^2^.

## Résultats

Deux cent deux (202) femmes ménopausées de plus de 50 ans ont été retenues pour participer à l’étude. Le pourcentage des femmes ostéoporotiques a été de 24.8%, celui des femmes ostéopéniques de 39.1%. 97.7% des femmes inclues ont déclaré ne pas avoir d'activité physique et 8% ont eu des fractures datant de plus de deux ans.

**Comparaison entre les groupes des ostéoporotiques, des ostéopéniques et des normaux (Tableau 1):** Par comparaison aux deux autres groupes, les femmes ostéoporotiques étaient plus âgées, plus anciennement ménopausées, présentaient un faible poids et un IMC bas. Les concentrations de la SHBG, la œ-CTX et l'OC ont été augmentées de manière significative dans ce groupe, alors que l'IEL était plus bas. Une hypovitaminose D, a néanmoins été objectivée dans les trois groupes.

**Tableau 1:** Comparaison entre les patients ostéoporotiques, ostéopénique et normaux (n = 202)

	Normal (n= 73)	Ostéopénie (n = 79)	ostéoporose (n = 50)	p	p_1_	p_2_	p_3_
**Age (année): m (ET)**	58.68 (7.28)	59.603 (7.71)	62.45 (7.72)	0.23	0.73	0.019	0.095
**Poids (kg): m (ET)**	81.20 (19.06)	78.06 (22.33)	67.34 (18.64)	0.001	0.608	0.001	0.011
**Taille (cm): m (ET)**	151.34 (24.66)	151.75 (18.73)	153.22 (13.58)	0.871	0.991	0.866	0.913
**IMC(kg/m** ^**2**^ **): m (ET)**	31.37 (4.2)	30.38 (4.7)	27.412 (5.5)	<0.001	0.39	<0.001	<0.001
AM **(année): m (ET)**	8.24 (8.385)	9.18 (8.474)	12.82 (8.92)	0.013	0.78	0.012	0.052
**SHBG (ng/ml): m (ET)**	45.31 (21.84)	57.53 (30.73)	65.78 (28.34)	<0.001	0.017	<0.001	0.217
β**-CTX(ng/ml): m (ET)**	0.389 (0.198)	0.41 (0.214)	0.522 (0.304)	0.007	0.84	0.007	0.042
**PTHi (pg/ml): m (ET)**	34.59 (24.64)	40.72 (31.55)	47.12 (45.829)	0.127	0.501	0.107	0.543
**E2(pmol/l): m (ET)**	39.7 (20.83)	36.21 (19.15)	33.52 (16.26)	0.202	0.498	0.186	0.719
**IEL(pmol/nmol): m (ET)**	11.61 (9.08)	8.58 (7.416)	6.78 (5.306)	0.0027	0.041	0.002	0.394
**S-DHEA(µmol/l): m (ET)**	2.36 (1.32)	1.923 (1.078)	1.97 (0.9)	0.46	0.052	0.159	0.969
**VITD(ng/ml): m (ET)**	11.469 (7.453)	13.472 (9.93)	11.32 (6.653)	0.232	0.303	0.995	0.33
**OC(µg/l): m (ET)**	22.11(10.964)	23.52 (12.21)	28.21 (17.14)	0.038	0.787	0.033	0.123
**DMO RL (g/cm** ^**2**^ **): m (ET)**	1.13 (0.12)	0.95 (0.23)	0.80 (0.058)	<0.001	<0.001	<0.001	<0.001
**Tscore RL: m (ET)**	-0.225 (1.009)	-1.433 (0.924)	-2.922 (0.49)	<0.001	<0.001	<0.001	<0.001
**DMOCF (g/cm** ^**2**^ **): m (ET)**	1.02(0.12)	0.918 (0.089)	0.913 (0.22)	<0.001	<0.001	<0.001	<0.001
**Tscore CF: m (ET)**	-0.027 (0.98)	-0.894 (0.759)	-1.771 (0.96)	<0.001	<0.001	<0.001	<0.001

Moyenne ± écart-type: m (ET) *: <0.05 **:<0.001

Analyse de variance à un facteur + Tests Post hoc (Bonferroni)

**p: ostéoporose- ostéopénie-normal p**_**1**_ **ostéopénie - normal p**_**2**_ **: ostéoporose - normal p**_**3:**_ **ostéoporose – ostéopénie**

**DMORL:** Densité minérale osseuse au rachis lombaire **DMOCF:** Densité minéral osseuse au col de fémur **IEL** Indice de l'estradiol libre **SHBG:**Sex Hormones Binding Globulin **PTH**_**i**_: Parathormone intacte **OC:** Osteocalcine β**-CTX:** Crosslaps **VitD**: 25-hydroxyvitamine D **IMC:** Indice de masse corporelle **E:** Estradiol AM: âge de la ménopause

**Corrélation entre la densité minérale osseuse, les hormones sexuelles et les marqueurs de remodelage osseux (Tableau 2) La DMO**: une corrélation négative a été établie entre la DMO au rachis lombaire et l’âge, l’âge de la ménopause, les taux sérique de la SHBG, de la œ-CTX et de l'OC. Il existait une corrélation positive entre la DMO au rachis lombaire et le poids, l'IMC et l'IEL. La DMO au col de fémur était positivement corrélée au poids, à l'IMC, à l'IEL et au taux de S-DHEA. Il existait, cependant, une corrélation négative de la DMO au col de fémur avec l’âge, l’âge de la ménopause d'une part et les taux de SHBG, de œ-CTX et d'OC d'autre part.

**Tableau 2:** Corrélation entre les hormones sexuelles, les marqueurs du remodelage osseux, âge, IMC et la densité minérale osseuse (n = 202)

	Age (année)	Poids (kg)	IMC (kg/m^2^)	AM (année)	SHBG (ng/ml)	β-CTX (ng/ml)	PTHi (pg/ml)	E2 (pmol/l)	IEL (pmol/nmol)	S-DHEA (µmol/l)	VITD (ng/ml)	OC (µg/l)
**Poids (kg)**	-0.1144											
**IMC (kg/m** ^**2**^ **)**	-0.0905	0.796**										
**AM (année)**	0.733**	-0.0805	-0.0519									
**SHBG (ng/ml)**	0.189**	-0.297**	-0.247**	0.146*								
β**-CTX (ng/ml)**	-0.0040	-0.0857	-0.152*	-0.0467	0.259**							
**PTHi (pg/ml)**	0.0332	0.0557	0.0131	0.0384	0.1341	0.307**						
**E**_**2**_ **(pmol/l)**	0.0821	0.324**	0.313**	0.0168	-0.0323	-0.0839	0.0965					
**IEL (pmol/nmol)**	-0.0677	0.443**	0.403**	-0.0875	-0.673**	-0.235**	-0.0203	0.723**				
**S-DHEA (µmol/l)**	-0.205**	0.1001	0.0715	-0.149*	-0.190**	-0.1267	-0.1271	0.237**	0.295**			
**VITD (ng/ml)**	-0.0471	-0.0969	-0.0484	0.0109	0.1176	-0.0992	-0.1357	0.0938	-0.0159	0.0546		
**OC (µg/l)**	-0.0366	-0.1057	-0.1281	-0.0708	0.276**	0.747**	0.310**	-0.157*	-0.305**	-0.206**	-0.1302	
**DMO RL (g/cm** ^**2**^ **)**	-0.223**	0.343**	0.288**	-0.288**	-0.320**	-0.170*	-0.0411	0.0873	0.293**	0.0298	-0.0049	-0.155*
**DMOCF (g/cm** ^**2**^ **)**	-0.339**	0.409**	0.424**	-0.366**	-0.395**	-0.230**	-0.0768	0.1208	0.374**	0.141*	0.0252	-0.270**

* <0.05

** <0.001

**Corrélation entre les hormones sexuelles et les marqueurs de remodelage osseux**: il existait une corrélation négative de l'E2 à l'OC et de l'IEL à la œ-CTX et l'OC (<0.001). La corrélation était positive entre le taux de S-DHEA et l'OC (<0.001) d'une part et la SHBG et la œ-CTX ou encore l'OC d'autre part (<0.001).

**Régression linéaire multiple (Tableau 3)**: en utilisant une régression linéaire multiple, les paramètres prédictifs de la DMO au rachis lombaire étaient le taux de SHBG et le poids. Les principaux paramètres prédictifs de la DMO au niveau du col fémoral étaient l’âge et l'IEL.

**Tableau 3:** Régression linéaire multiple avec la densité minérale osseuse comme variable indépendant (n = 202)

	Col de fémur		
	coefficient	corrélation partielle	p
**Age (Année)**	-0.0080	-0.2791	0.0001
**IEL(pmol/nmol)**	0.0052	0.1908	0.0072
		**Rachis lombaire**	
	**coefficient**	**corrélation partielle**	**p**
**SHBG (ng/ml)**	-0.0016	-0.2177	0.0021
**Poids (Kg)**	0.0020	0.2093	0.0032

## Discussion

Au terme de cette étude, chez les femmes ménopausées d'origine marocaine et sans pathologies associées aux perturbations du remodelage osseux, les paramètres prédictifs indépendants de la DMO respectivement au col de fémur et au rachis lombaire sont représentés par le taux sérique de la SHBG et le poids d'une part ainsi que l’âge et l'IEL d'autre part.

Dans le présent travail, la prévalence de l'ostéoporose était de 25%, plus faible que celles rapportées dans d'autres études marocaines récentes [7–9]. Cette différence pourrait s'expliquer par la petitesse de la cohorte étudiée ou encore le type de l’étude. 97.7% des femmes inclues dans la présente étude ont déclaré ne pas avoir d'activité physique. L'augmentation dramatique des fractures du col de fémur dans les quarante dernières années pourrait être en rapport avec l'augmentation de l'espérance de vie, mais semble également liée à la sédentarité. De plus, au cours de la ménopause les perturbations hormonales accélèrent la perte osseuse qui sera accentuée par l'absence de l'activité physique régulière [10, 11].

Dans la série étudiée, les femmes ostéoporotiques ont été plus âgées par rapport au groupe des femmes normales. L’âge a, en effet, été négativement corrélé à la DMO au col de fémur et au rachis lombaire. La DMO diminue avec l´âge, et de façon plus prononcée après la ménopause [12]. Il a été rapporté qu'après l’âge de 50 ans, la fréquence des fractures du col de fémur est deux fois plus importante chez la femme par rapport à l'homme [13]. Un des facteurs importants de risque d'ostéoporose associé à une déficience en estrogènes chez les femmes est l’âge de la ménopause. L’âge de la ménopause était de 12.82 ans dans le groupe des femmes ostéoporotique et moins de 10 ans chez les femmes ostéopéniques et celles normales. Selon Ide et al. [14], une augmentation importante l'Odds ratio (OR) basée sur l’âge de la ménopause (dates des dernières règles) a été observée dans la tranche située entre 7-13 ans (OR = 2.3, IC 95%: 1.0 à 5.4) comparativement à celle de moins de 7 ans. En outre, plusieurs études ont montré une diminution significative de la DMO dans les 10 à 16 premières années après la ménopause [15–17]. L'hypovitaminose D objectivée dans les trois groupes (ostéoporose, ostéopenie, normal) sans différence significative, a également été rapporté dans l’étude précédente [9]. Sa forte prévalence pourrait être due au style vestimentaire, la majorité des patientes recrutées étaient en effet voilées, mais également aux habitudes des pays chauds d’éviter l'exposition au soleil, et l'absence de supplémentation vitaminique des produits alimentaires au Maroc.

Dans le présent travail, contrairement au taux sérique d’œstradiol, l'IEL, qui représente l'indice de l'estradiol libre ou biodisponible, affiche une différence très significative entre le groupe normal et le celui des femmes ostéoporotiques. Le taux sérique d’œstradiol était négativement corrélée à l'ostéocalcine, alors que l'IEL l’était de façon très significative aux taux sériques de la β-CTX et de l'OC (<0.001). De ce fait, la diminution du taux de l'oestradiol libre est associée à une augmentation du remodelage osseux. L'IEL représente un des paramètres prédictifs indépendants de la DMO au col de fémur par analyse multivariée. La diminution de l'estradiol biodisponible en fonction de l’âge peut donc expliquer en partie la diminution de la DMO au col de fémur. Greendale et al [18] ont également trouvé une corrélation négative et significative entre l’œstradiol et les marqueurs du remodelage osseux.

Les résultats de cette étude ne montrent aucune corrélation entre la DMO et l’œstradiol, et sont concordants avec ceux d'une étude chinoise [19]. Par contre, d'autres travaux ont objectivé des corrélations positives. Ainsi, Dans une étude portant sur 370 femmes ménopausées âgées de 55 à 85 ans, Van Geel et al [20] ont trouvé une association positive et significative entre le taux sérique d´œstradiol et la DMO au rachis lombaire et au col de fémur. Zarrabeitia et al ont révélé une corrélation significative entre le taux sérique d´œstradiol et la DMO au rachis lombaire chez les femmes ménopausées [21]. La corrélation positive objectivée entre l'IEL et la DMO au col de fémur et au rachis lombaire a également été rapportée par Lambrinoudaki et al et Gennari et al [22, 23]. Elle relie la diminution de l’œstradiol bio disponible à une diminution de la DMO.

Par ailleurs, nous avons révélé une association faiblement positive entre le taux du S-DHEA et la DMO au col de fémur, et une corrélation négative entre le taux sérique de la S-DHEA et l'ostéocalcine. Une corrélation positive significative entre la DMO et la S-DHEA a été notée chez 120 femmes ménopausées (51-99 ans) [19]. Szathmári et al [24] ont objectivé une relation positive entre le taux sérique de la S-DHEA et la DMO au col de fémur et au rachis lombaire chez 105 femmes âgées de 45-69 ans, 76 ménopausées et 29 femmes pré-ménopausées. En revanche, d'autres études n´ont trouvé aucune association entre le taux sérique de la S-DHEA et la DMO [25, 26]. La raison de cette différence pourrait être due à la taille de la population inclue dans l’étude, le type de l’étude et leur durée relativement courte. Dans la série étudiée, le taux sérique de la SHBG a été positivement et fortement associé aux marqueurs de résorption et de formation osseuse à savoir la β-CTX et l'OC (<0.001) respectivement. L'augmentation du taux sérique de la SHBG est de ce fait associée à une augmentation du remodelage osseux. Notons de plus que le taux sérique moyen de la SHBG dans notre étude est plus élevé dans le groupe ostéoporose par rapport au groupe normal et ce de manière significative. Les études prospectives réalisées au sein des cohortes EPIDOS (EPIdémiologie De l'Ostéoporose), OFELY (Os des Femmes de Lyon) et SOF (Study of Osteoporotic Fractures) ont montré une faible corrélation entre le taux sérique de la SHBG et les marqueurs de résorption chez la femme ménopausée [27–29]. Une étude a également montré une faible association entre un marqueur de formation osseuse, l'OC et le taux sérique de la SHBG [23].

Dans le présent travail, le taux sérique de la SHBG a été négativement corrélé à la DMO au col de fémur et au rachis lombaire, et représentait un des paramètres prédictifs indépendants de la DMO au rachis lombaire. Ces résultats ont été confirmés par d'autres études qui ont trouvé une corrélation négative entre le taux sérique de la SHBG et la DMO à l'extrémité supérieure du fémur, au trochanter et au rachis lombaire [30, 31]. Ainsi, plusieurs facteurs ont été identifiés comme affectant la production de la SHBG. Le sexe féminin est associé à des taux élevés de la SHBG [32]. Toute augmentation du poids est associée à une diminution du taux sérique de la SHBG, tandis que l'augmentation de l’âge est associée à son augmentation. Et puisque le taux sérique de la SHBG a un impact direct sur les hormones sexuelles biodisponibles, dans notre cas l’œstradiol, il va influencer directement la concentration de l’œstradiol bio disponible [33–35]. Nos résultats confirment qu'un taux sérique élevé de la SHBG est associé à une diminution de la densité minérale osseuse au col de fémur et au rachis lombaire et à une augmentation de la perte osseuse et logiquement à un risque élevé de fracture d'origine ostéoporotique [36]. La présente étude a des forces et des limites. Toutes les patientes qui ont participé à l’étude ne souffraient d'aucune maladie, ne fumaient pas, et ne prenaient aucun traitement pouvant perturber le métabolisme osseux. La partie pré-analytique était bien maitrisée, les analyses étaient effectuées le même jour et par le même technicien et dans le même laboratoire et selon les bonnes pratiques de la réalisation des analyses de biologie médicales. Toutes les mesures de la DMO par la technique d'absorptiométrie biphotonique à rayons X ont été réalisées avec un seul appareil de la densitomètrie osseuse bien contrôlé. Les principales limites sont inhérentes au type de l’étude (transversale) et à la procédure de recrutement des patientes qui étaient toues des bénévoles.

## Conclusion

En conclusion, cette étude a montré que l'augmentation de l’âge et la diminution de l’œstradiol libre expliquaient la diminution de la DMO au niveau du col de fémur. L'augmentation du taux sérique de la SHBG et la diminution du poids expliquaient la diminution de la DMO au rachis lombaire. De même, la diminution significative de l’œstradiol bio disponible est associée à une diminution de la DMO au rachis lombaire et au col de fémur. Cette diminution est associée et de manière très significative à une augmentation des marqueurs de remodelage osseux de la formation et de la résorption. La SHBG a été associée à une diminution de la DMO au rachis lombaire et au col de fémur. Cette diminution est également corrélée et de manière très significative à une augmentation des marqueurs de remodelage osseux de la formation et de la résorption.
